# Extracellular Vesicles From Young Human Myogenic Progenitor Cells Rejuvenate Aged Cells

**DOI:** 10.1155/bri/5967706

**Published:** 2026-06-28

**Authors:** Lorenzo Marramiero, Ester Sara Di Filippo, Federica Di Marco, Piero Del Boccio, Cristian Celia, Luisa Di Marzio, Nicola d’Avanzo, Tiziana Pietrangelo, Stefania Fulle, Rosa Mancinelli

**Affiliations:** ^1^ Dept Neuroscience, Imaging and Clinical Sciences, “G. d’Annunzio” University Chieti-Pescara, 66100, Chieti, Italy, unich.it; ^2^ Interuniversity Institute of Myology, 06132, Perugia, Italy, coram-iim.it; ^3^ Functional Evaluation Laboratory, “G. d’Annunzio” University Chieti-Pescara, 66100, Chieti, Italy, unich.it; ^4^ Dept Sciences, “G. D’Annunzio” University Chieti-Pescara, Chieti, 66100, Italy, unich.it; ^5^ Dept Pharmacy, “G. D’Annunzio” University Chieti-Pescara, Chieti, 66100, Italy, unich.it; ^6^ Department of Experimental and Clinical Medicine, “Magna Graecia” University of Catanzaro, Catanzaro, 88100, Italy, unicz.it; ^7^ Research Center “ProHealth Translational Hub”, Department of Experimental and Clinical Medicine, “Magna Graecia” University of Catanzaro, Catanzaro, 88100, Italy, unicz.it

**Keywords:** extracellular vesicles, human adult myogenic progenitor cells, microRNAs, senescence, skeletal muscle

## Abstract

The physiological age‐related decline in skeletal muscle mass, power, and function is challenging for humans. Skeletal muscle has been recently recognized as a secretory organ, with human myogenic progenitor cells (hMPCs) releasing extracellular vesicles (EVs). Here, we investigate the role of hMPC‐derived EVs as mediators in skeletal muscle aging. This heterologous approach enables the analysis of age‐related variations in EV burden and their impact on human muscle stem cell function. Therefore, we isolated EVs from hMPCs obtained from *vastus lateralis* muscle biopsies of young and elderly subjects. Then, we characterized EVs for specific marker, size, and concentration and analyzed their miRNA expression and proteomic profiles to delineate the bioactive cargo that influences recipient cell signaling. Next, we tested the ability of EVs to modulate on hMPCs. Specifically, we treated elderly hMPCs with young EVs and vice versa to analyze viability and differentiation. Our results demonstrate that EVs released by young hMPCs carry regenerative signals that mitigate the functional decline of aged muscle stem cells. Conversely, the EVs derived from elderly hMPCs compromise the regenerative capacity of their younger counterparts. Therefore, these results suggest that hMPCs release EVs and that their cargo is modulated by donor age. Moreover, the EVs significantly modulated hMPCs’ viability and differentiation in cell culture.

## 1. Introduction

Elderly people typically experience an age‐specific muscle aging. It is characterized by a progressive loss of muscular mass and strength, reduction in muscle regenerative abilities, and susceptibility to muscular pathologies [1–3]. Muscle aging is a multifactorial process heavily driven by the declining activity of muscle stem cells, known as satellite cells (SCs). Upon injury or damage, these cells activate and differentiate into human myogenic progenitor cells (hMPCs) to mediate muscle regeneration [4–6]. SCs are responsible for the postnatal growth of skeletal muscle tissue, the maintenance of muscular mass in adulthood, and tissue regeneration in case of muscle damage. SCs are located beneath the basal lamina surrounding each myofiber and are normally quiescent; in case of damage, they become rapidly activated, migrate, proliferate, and fuse with the damaged myofibers to repair them and/or with each other to form new myofibers. This process is called myogenesis. However, a fraction of mitotically arrested SCs do not differentiate into fusion competent myocytes and return to a quiescent status, thus reconstituting the pool of quiescent SCs [7].

Activation and proliferation stages of SCs and subsequent myogenesis are characterized by the expression of myogenic regulatory factors, and the phases of this process typically involve the sequential expression of proteins, including Pax3 (quiescent SCs), myoD (proliferating myogenic progenitor cells), and myogenin (differentiated myoblasts) [8]. Myogenesis in turn can be regulated by muscle‐specific microRNAs (miRNAs), myomiRs (miR‐1, miR‐133a and b, and miR‐206), and small non‐coding RNAs that bind to mRNA targets and interfere with translation of RNA into protein synthesis [9, 10].

Alongside its classic functions, skeletal muscle has been recently identified as a secretory organ capable of producing and releasing extracellular vesicles (EVs), from both fibers and hMPCs [11, 12]. EVs are small spherical structures, classified by average sizes that contain a variety of bioactive molecules, including proteins and miRNAs [13]. Indeed, due to their cargo of bioactive molecules, EVs can deeply affect the behavior of recipient cells that can be parent cells and distant tissues. Both the production and cargo of EVs generated by hMPCs are affected by various physiological factors such as age, lifestyle, and sports [14, 15]. To date, several papers aimed to identify intrinsic and extrinsic factors that contribute to explain the different proliferative and regenerative properties between young and elderly hMPCs. Accordingly, our previous works demonstrated that young and elderly hMPCs exhibit similar myogenicity in vitro. However, elderly hMPCs struggle to complete the differentiation program compared to younger ones. This impairment is driven by increased oxidative stress, reduced antioxidant enzyme activity, altered gene expression patterns, higher susceptibility to apoptosis, and dysregulated myomiR expression [16–19].

In addition, young and aged SCs are able to condition their own behavior via an autocrine mechanism differentially releasing promyogenic factors both in vivo in the niche and in vitro in the culture media [20, 21]. Indeed, it was demonstrated that the treatment of elderly hMPCs with young hMPC‐conditioned medium enhanced elderly hMPCs’ proliferation and their subsequent differentiation [22]. Therefore, the aim of this work was to investigate the specific contribution due to EVs released in the media by hMPC cultures on cell behavior. In detail, we isolated, characterized, and analyzed myomiRs and proteomic profile of EVs collected from both young and elderly hMPC cell culture media and then we studied the effects of EVs released by hMPC cultures collected from young subjects on viability and regenerative properties of hMPCs derived from elderly subjects and vice versa.

## 2. Materials and Methods

### 2.1. Cell Cultures

hMPCs were obtained from *vastus lateralis* skeletal muscle biopsies following the procedure described by Fulle et al. [16] by tiny percutaneous needle biopsy [23]. Young (26.5 ± 1.65 years; *n* = 10) and elderly (68.86 ± 1.35 years; *n* = 8) healthy untrained male subjects underwent voluntary biopsies.

Exclusion criteria were as follows: irregular ECG, osteoarticular pathologies, mild‐to‐medium cardiocirculatory pathologies, diabetes type I or II, not controlled hypertension, cancer, neurological or psychiatric disease, respiratory pathology, neuromuscular disease, and genetic disease. All participants provided their written informed consent.

To obtain hMPCs, muscle biopsies were processed using the method of explants previously described [23, 24]. The cells were cultured for growth and differentiation according to Di Filippo et al. [25]. hMPCs were grown at 37°C in 5% CO_2_ humidity, in growth medium (GM): HAM’s Nutrient Mixture F10 without L‐glutamine (#ECB7503L, Euroclone, Milan, Italy) supplemented with 20% fetal bovine serum defined (FBS; Hyclone, #SH30070.03, Cytiva, USA), 1% penicillin–streptomycin solution 100X (#ECB3001D, Euroclone), 50 μg/mL gentamicin (#ECM0011D, Euroclone), and 1% stable glutamine (200 mM) (#ECB3004D, Euroclone).

To induce differentiation into myotubes, hMPCs were added to a differentiation medium (DM), composed of Dulbecco’s Modified Eagle’s Medium high‐glucose (#ECB7501L, Euroclone) supplemented with 5% heat‐inactivated horse serum (#ECS0091L, Euroclone), 50 μg/mL gentamicin, 1% stable glutamine (200 mM), 1% penicillin–streptomycin solution 100X, 10 μg/mL insulin solution from bovine pancreas (#I0516‐5 mL, Sigma‐Aldrich, St. Louis, MO, USA), and 100 μg/mL of apo‐transferrin human (#T2036, Sigma‐Aldrich, St. Louis, MO, USA). We followed the differentiation process until 7 days.

### 2.2. Characterization of hMPCs

To characterize hMPCs for myogenic purity and differentiation efficiency, immunocytochemical assays for desmin and myosin heavy chain were performed, respectively, following the protocol described by Di Filippo et al. [19]. The cells were treated with one of the following primary antibodies: monoclonal mouse primary anti‐human desmin, clone D33 (#M0760, DAKO, USA), or monoclonal mouse primary antibody anti‐myosin heavy chain, clone MF20 (Developmental Studies Hybridoma Bank, University of Iowa, USA). Myogenic cells and differentiated cells were identified by brown staining (#K5001, DAKO, USA), with hematoxylin used for counterstaining.

Image acquisition consisted of 10 random fields per well, captured with a digital camera (Canon EOS 350D, Japan) connected to the microscope (Leica Microsystems CMS GmbH, Germany) at 20x magnification. Subsequently, desmin‐positive cells were counted using the ImageJ software, and the percentage of myogenic purity was calculated as the ratio of the number of desmin‐positive cells to the total number of cells multiplied by 100. Instead, the differentiation rate was calculated as fusion index percentage (FI%) as the ratio of the number of nuclei in the myotubes positive for myosin heavy chain to the total number of nuclei multiplied by 100. Furthermore, for each sample, the analysis of stained area was performed using Immunohistochemistry Image Analysis Toolbox, ImageJ software.

In this study, all samples used were at least 60% desmin‐positive cells (data not shown).

### 2.3. Isolation and Purification of EVs From Cell Culture Media

Cell culture media were collected and measured from proliferating young and elderly hMPCs at 80%–90% confluence, coinciding with cell harvesting. Subsequent characterization data were then normalized to the final cell count determined at the time of cell harvesting and on cell culture volume collected (Table 1).

**TABLE 1:** Particle concentration of EVs based on average sizes and number of young and elderly subjects collected during the analysis.

	**Size (nm)**	**SD**	***N***	**EVs/mL**	***N***	**EVs/cell**	***N***

Elderly EVs	2.44 × 10^2^	1.58 × 10^2^	13	4.94 × 10^9^	12	5.31 × 10^4^	12
Young EVs	2.59 × 10^2^	1.48 × 10^2^	17	6.62 × 10^9^	19	1.70 × 10^5^	15

Subsequently, EVs were purified from the collected media following the method published by our group [26]. Moreover, we performed the same step of isolation also for the FBS Hyclone, used to grow the hMPCs, in order to demonstrate that the EVs are not derived from the serum but from the samples. Furthermore, as reported in the manufacturer instruction, the FBS Hyclone used is 40‐nm‐filtered. All centrifugation steps were performed with the Optima XL‐100 K ultracentrifuge, rotor SW 28 Swinging‐Bucket Rotor (Beckman Coulter, USA). The resulting pellet was resuspended in 50 μL of Dulbecco’s phosphate buffer saline (PBS) w/o calcium, w/o magnesium (PBS, #ECB4004L, Euroclone, Milan, Italy), for later particle size, size distribution, shape, and zeta‐potential analysis, along with molecular analyses.

### 2.4. Treatment: hMPCs With EVs

In order to evaluate the viability and differentiation, we treated elderly hMPCs with young EVs and vice versa.

In detail, elderly and young hMPCs were seeded in GM (1600 cells per well) in 96‐well plates and, after 24 h, were treated with young or elderly EVs at the concentration of 1 × 10^7^, 5 × 10^6^, and 2.5 × 10^6^ EVs/well. After EV incubation of 24, 48, and 72 h, the hMPCs’ viability was valuated using the MTT assay.

To assess the differentiation process, elderly and young hMPCs were seeded in GM (5300 cells per well) in 48‐well plates; after 48 h, at reaching the confluence, the GM was changed with DM and young or elderly EVs were added at the concentration of 1 × 10^7^, 5 × 10^6^, and 2.5 × 10^6^ EVs/well. After 7 days, the immunofluorescence assay was performed. hMPCs cultured both in GM and DM without any treatment with EVs were used as controls.

The choice of EVs’ concentration was guided by quantification data (see Table 1).

### 2.5. Western Blotting (WB) for EV Identification

WB analysis was performed both on young and elderly EVs and also on FBS Hyclone after isolation procedure. WB was performed following the protocol described by Pietrangelo et al. [26]. Briefly, the EVs were lysed with Exosome Resuspension Buffer (Thermo Fisher Scientific, Cat # 4478545), denatured, loaded onto BoltTM 4%–12% Bis‐Tris Plus Gels (Invitrogen REF NW04120BOX), and transferred to nitrocellulose membrane with iBlot 2 Dry Blotting System (Thermo Fisher Scientific, Cat # IB21001). Membrane was incubated with primary antibodies, CD81 (1.3.3.22, sc‐7637, Santa Cruz Biotechnology, Inc.) at 1:200, and secondary HRP‐conjugated antibodies (Cell Signaling Technology) at 1:5000. Bands were detected at UVITEC machine (Cambridge) by LiteAblot PLUS enhanced chemiluminescent substrate (Euroclone).

### 2.6. Immunofluorescence Analysis for EV Characterization

EVs were seeded onto coverslip slides and allowed to adhere for 24 h. Subsequently, the samples were fixed with 4% of paraformaldehyde (Polysciences, Valley Road Warrington, USA) for 15 min in PBS, permeabilized with 0.2% Triton X‐100 in PBS containing 1% (w/v) of bovine serum albumin, and blocked with 1:10 donkey serum (all from Sigma‐Aldrich, St. Louis, MO, USA) for 10 min. EVs were incubated overnight at 4°C with the primary anti‐Alix antibody diluted 1:100 (#MA1‐83977, Invitrogen, Life Technologies, Waltham, MA, USA). Subsequently, EVs were incubated for 45 min with Alexa Fluor 488‐conjugated secondary antibody diluted 1:2000 (#A‐21202, Invitrogen, Life Technologies, Waltham, MA, USA). Negative control was performed with the same protocol without primary antibody (anti‐Alix). Images were acquired with a Zeiss LSM800 (Carl Zeiss, Jena, Germany) confocal microscope with a 63x objective lens and 4x digital zoom, in confocal mode. Scanning along the *Z*‐axis was performed with a step size of 0.02 μm. Images were analyzed by ZEN 3.8 software.

### 2.7. Dynamic Light Scattering and Zeta Potential

EVs were physicochemically characterized for average sizes and particle concentration by using Zetasizer Ultra (Malvern Instruments Ltd, Malvern, UK) as previously reported [27]. Briefly, samples were suitably diluted (1:20 v/v) in an isotonic buffer prefiltered through 0.22‐μm polypropylene membranes (Whatman Inc., Clifton, NJ, USA) to prevent multiple scattering. The solutions were then placed in disposable sizing cuvettes (Malvern Instruments Ltd, Malvern, UK) for analysis at 25°C. A backscattering detection angle of 173° was used for the detection of average sizes and particle concentration, and the following parameters were set up: real refractive index of 1.59, imaginary refractive index of 0.01, medium refractive index of 1.330, medium viscosity of 1.0 mPa s, and medium dielectric constant of 80.4. All measurements for average sizes and particle concentration are three independent measurements for each sample ± standard deviation (S.D.).

### 2.8. Total RNA Isolation From EVs

Total RNA from EVs was isolated using the Total Exosome RNA and Protein Isolation Kit (Catalog Number 4478545; Invitrogen, Life Technologies) according to manufacturer’s instruction; the quantification of total RNA was performed using NanoPhotometer NP80 (IMPLEN).

### 2.9. EVs’ miRNA Expression Profile

Retrotranscription of 20 ng of total RNA was carried out according to the Applied Biosystems’ High Capacity cDNA Reverse Transcription kit (#4368814, Applied Biosystems, Life Technologies, Monza, Italy). Quantitative real‐time PCR was performed using the TaqMan probes and the specific TaqMan Universal Master Mix II, no UNG, in 96‐well plates (#4440040, Applied Biosystems, Life Technologies, Monza, Italy) with a QuantStudio 7 Pro Real‐Time PCR System. The ubiquitous hsa‐miR‐16‐5p (#000391) was used as an internal control. miR‐16 has been identified as a candidate control gene showing the least variability in cell lines from Applied Biosystems (Application note TaqMan MicroRNA Assays). The specific miRNA sequence probes used (Applied Biosystems) were hsa‐miR‐133a (#002246), hsa‐miR‐133b (#002247), hsa‐miR‐1 (#002222), and hsa‐miR‐206 (#000510).

### 2.10. EVs’ Proteomic Profile

Proteomics analysis was performed by using the same number of EVs in the two experimental conditions: 70.000.000 EVs from both elderly and young subjects. As reported in our previous work [28], EV lysates were digested by trypsin (Trypsin, Sigma‐Aldrich, St. Louis, MI, USA) according to the Filter‐Aided Sample Preparation (FASP) protocol, and the digested proteins were acquired in triplicate by LC–MS/MS using the UltiMateTM 3000 UPLC (Thermo Fisher Scientific, Waltham, MA, USA) chromatographic system coupled to the Orbitrap FusionTM TribridTM (Thermo Fisher Scientific, Waltham, MA, USA) mass spectrometer with an EASY‐spray AcclaimTM PepMapTM C18 (75 μm ID, 15 cm L, 2 μm PS, Thermo Fisher Scientific) nanoscale chromatographic column. Proteomic data were acquired and processed according to Di Stefano et al. [29] by using Proteome Discoverer Version 2.4.0.305 (Thermo Fischer Scientific) and the sequence database *Homo sapiens* (SwissProt TaxID = 9606) FASTA. Univariate statistical analysis was performed to individuate differentially expressed proteins (DEPs) between two experimental conditions through “Protein Abundance Ratio” values for each quantified proteins, which were uploaded to Ingenuity Pathway Analysis tool (IPA, Qiagen, Hilden, Germany) to perform a Gene Ontology and functional enrichment analysis, mapping statistically DEPs to their functional annotation such as “canonical pathways” analysis, “upstream regulators” analysis, and “disease and function effects” networks based on the comparison of our protein dataset with published literature. Briefly, as reported in [30], IPA returns the results by assigning to each pathway a value of ‐log 10 (*p* value) and a z‐score value. The ‐log 10 (*p* value) measures the statistical overlap between the protein dataset and the functional categories; the significance is attributed to −log10 (*p* value) greater than 1.3. Instead, the predicted modulation of each “canonical pathway,” “upstream regulator,” and “downstream” (Supporting File 1) network effect is inferred by the z‐score generated by the IPA system (z‐score ≥ 2.0 means that a molecule or pathway is predicted to be activated, whereas z‐score ≤ −2.0 means the predicted inhibition of target molecules or pathways). In this context, each identified DEP and its corresponding “Protein Abundance Ratio” were uploaded as a global matrix into the IPA tool to proceed with functional analysis based on protein expression values. The mass spectrometry proteomic data have been deposited to the ProteomeXchange Consortium via the PRIDE partner repository with the dataset identifier PXD063794.

### 2.11. Viability of huMPCs After EV Treatment by MTT Assay

To perform the assay, huMPCs (1600 cells per well) were seeded in 96‐well plate, and after 24 h, the cells were treated with 1 × 10^7^, 5 × 10^6^, and 2.5 × 10^6^ of EVs for 24, 48, and 72 h. MTT assay was performed following the protocol described by Purcaro et al. [31]. Briefly, at each time point, cellular viability was evaluated by incubating cell cultures in 20 μL of 3‐(4,5‐dimethyl‐2‐thiazolyl)‐2,5‐diphenyl‐2H‐tetrazolium bromide at a concentration of 5 mg/mL (#475989, Sigma‐Aldrich, USA) solution to each well, held for 3 h in incubator at 37°C in the dark. Thereafter, the plates were centrifuged at 2000 rpm for 15 min. Then, the supernatant was removed, 200 μL of dimethyl sulfoxide (#D5879, Sigma‐Aldrich, USA) was added to each well, and the plates were incubated for 30 min at 37°C in the dark. The absorbance was determined by spectrophotometry (Synergy H1 BioTek) at a wavelength of 540 nm.

### 2.12. Statistical Analysis

Statistical analysis was performed with unpaired *t*‐test in order to identify statistical differences, with a *p* value ≤ 0.05 considered statistically significant, using the software GraphPad Prism, Version 9.3.1 (GraphPad Software, USA). For miRNA expression and EV size and concentration, the young group was compared to the elderly group; for cell viability and fusion index, cells stimulated with EVs were compared with control cells without EV treatment.

## 3. Results

### 3.1. WB for EV Identification

**FIGURE 1:**
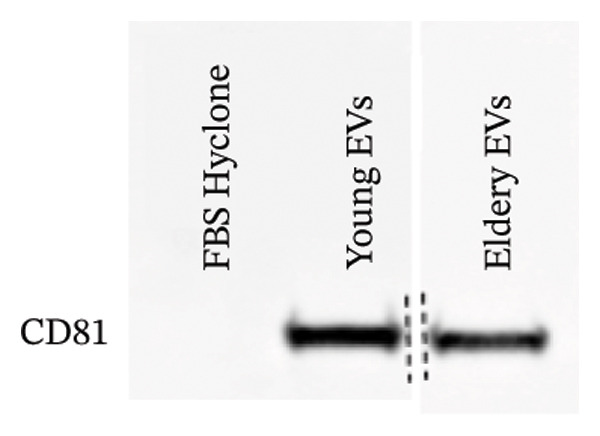
CD81 protein expression. Western blotting analysis was performed on young and elderly EVs and FBS Hyclone. The bands were taken from two nonadjacent lanes originating from exactly the same gel and blot with exactly the same exposure time, but spliced together indicated with double‐dotted lines.

The expression of CD81, a specific marker for EVs [32], was assessed in EVs isolated from the GM of young and elderly subjects, as well as in FBS Hyclone. Our results confirmed the presence of CD81 in EVs derived from both age groups, whereas it was undetectable in the FBS Hyclone used as a medium component. Representative CD81 bands are shown in Figure 1.

### 3.2. EVs’ Physicochemical Characterization

**FIGURE 2:**
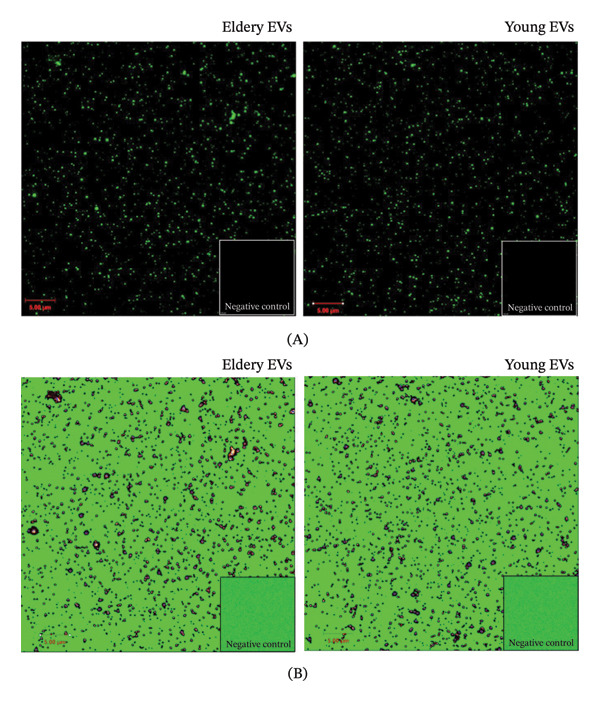
Immunofluorescence staining for ALIX (green) marker on elderly and young EVs. (A) Elderly and young EVs acquired for Alexa 488. (B) Elderly and young EVs’ corresponding range indicator mode analysis of the same fields, displaying pixel intensity distribution and signal saturation assessment. Negative control: elderly and young EVs without anti‐ALIX antibody. Images were acquired with ZEN software (Zeiss). Signal saturation (red) and pixels off (green). Scale bar: 5 μm. Representative images of three independent experiments. The signal‐free negative control in both samples excludes background noise, ensuring the validity of the comparison.

In order to confirm the presence of exosomal markers in our samples, we tested the ALIX marker protein for elderly and young EVs. We observed that the ALIX protein was present and localized in both young and elderly EVs. Confocal microscopy analysis revealed robust ALIX immunosignal (Alexa Fluor 488, green channel) distributed throughout EV populations from both age groups (Figure 2A,B). Qualitative morphological analysis confirmed that ALIX localization was consistent with exosomal architecture, revealing spherical structures with heterogeneous signal intensity distribution across individual EVs.

**FIGURE 3:**
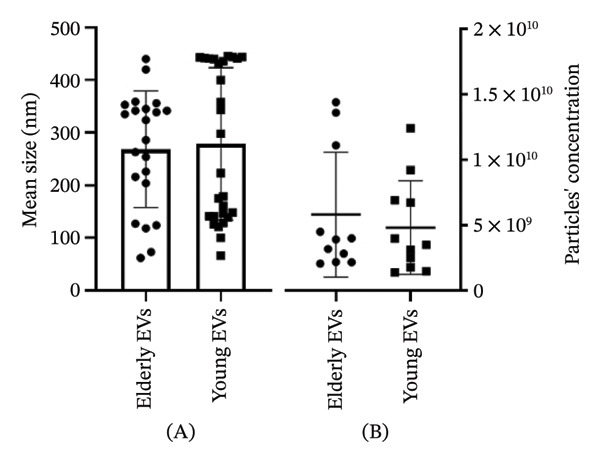
Physicochemical characterization of EVs. Average size (A) and particle concentration (B) were measured at 25°C by using Zetasizer Ultra apparatus. Three different measurements were carried out for each sample, and results are the average ± S.D. We analyzed elderly (*n* = 8) and young (*n* = 10) EVs.

EVs had average sizes of 279 ± 145 (young EVs) and 268 ± 111 (elderly EVs) nm, while the particle concentration was 4.81E+09 ± 3.57E+09 (young EVs) and 5.8E+09 ± 4.76E+09 (elderly EVs) (Figure 3). EVs, that have been collected by young subjects, are better size distributed than EVs that have been collected by elderly subjects (Figure 3A). Moreover, the average size is similar between two samples, and no significant differences have been carried out after the isolation and subsequent characterization (Figure 3A). The particle concentration of EVs is over *E*+09 for both young and elderly subjects, and many particles, with the same average sizes, are distributed in the collected samples in similar range of concentration (Figure 3B). The particle concentrations of EVs, collected by young subjects, showed that they are more narrow size distributed than EVs, collected by elderly subjects (Figure 3B), and these data were in agreement with the average sizes of EVs that had similar scale for both collected samples (Figure 3A).

### 3.3. EVs’ Quantification and Characterization

Using the DLS NanoZS Ultra, we quantified the EVs released from the culture media of young and elderly hMPCs. Normalizing the data to the total cell count in cultured and on the volume collected, a significantly higher average number of EVs per cell was observed in young compared to elderly samples (1.70 × 10^5^ with respect to 5.31 × 10^4^) (Table 1). This finding is a key observation, as it suggests that young cells not only have a different load as further discussed but also actively produce more EVs.

### 3.4. EVs’ miRNA Profile

**FIGURE 4:**
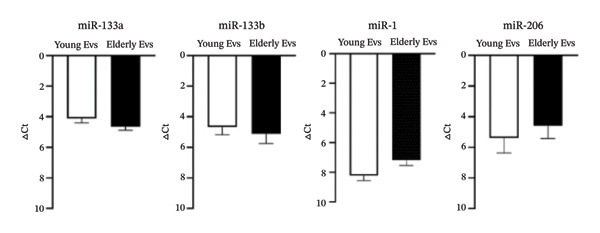
miRNA’s expression analysis on young and elderly EVs. The expression levels of miR‐133a, ‐133b, ‐1, and ‐206 were analyzed in EVs derived from young and elderly hMPC culture media. The ubiquitous miR‐16 was used as housekeeping gene to normalize miRNA levels. Three independent experiments were performed in triplicates, and results are shown as ΔCt (Ct_miRNA of interest_‐Ct_miR-16_). Data are expressed as mean ± SEM.

Expression analysis of miRNAs‐133a, ‐133b, ‐1, and ‐206 was performed on young and elderly EVs isolated from proliferating cell culture media. Analysis of the miRNA expression from young and elderly EVs showed a trend of upregulation of both miR‐1 and miR‐206, while miR‐133a and miR‐133b expression showed a trend of downregulation in elderly versus young EVs (Figure 4).

### 3.5. EVs’ Proteomic Profile

**FIGURE 5:**
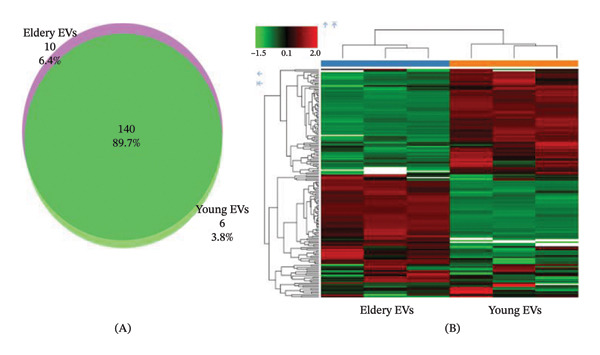
Venn diagram and heatmap. (A) The Venn diagram shows the unique and shared proteins robustly quantified in our samples of young and elderly EV subjects. (B) The heatmap describes the protein expression trend in the two experimental conditions, elderly and young EVs: as it is possible to note, all analytical replicates (*n* = 3) for each condition (*n* = 2) correctly cluster apart.

By purified EVs from proliferating cell culture media of young and elderly subjects, we quantified a total of 156 proteins. Excel file “Proteomics report” in the Supporting File 2 contains the list of the quantified proteins common in at least two of the analyzed replicates (*n* = 3). The Venn diagram in Figure 5A shows the unique and shared proteins identified across the two experimental conditions: young and elderly EVs. Among the 156 quantified proteins, 89.7% of them are present in both groups, while 6.4% and 3.8% are unique proteins in, respectively, elderly EVs and young EVs. The heatmap in Figure 5B shows the protein abundances related to each analytical replicate of two experimental conditions, correctly clustered within elderly EV and young EV samples.

**FIGURE 6:**
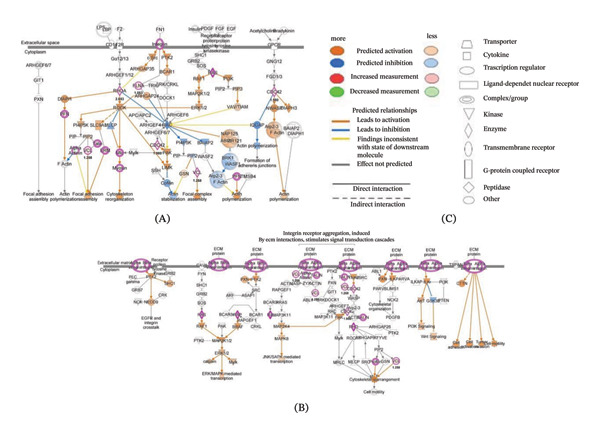
Protein network comparing young EVs versus elderly EVs. (A, B) Upregulated canonical pathway networks predicted by IPA functional enrichment analysis: “Actin cytoskeleton signaling” and “Integrin signaling.” (C) The IPA network’s legend describes the bubble and line’s colors and the line’s hatchings to better understand the results. In particular, the orange and blue bubbles indicate, respectively, the predicted activation and inhibition of canonical pathways, upstream regulator, and disease and biofunctions; on the other hand, red and green bubbles, respectively, show the increased and decreased protein’s measurement. Purple bubbles represent a specific “protein family name.” The dotted and not dotted network’s lines describe the direct and indirect interaction between molecule–molecule and molecule–function; these lines’ colors indicate a connection with pathways or molecules’ activation (orange lines), inhibition (blue lines), inconsistent findings (yellow lines), and not predicted effects (gray lines), always based on literature data.

**FIGURE 7:**
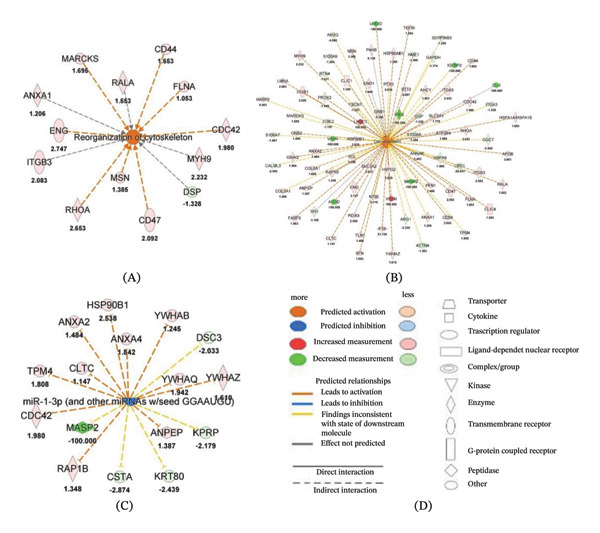
Protein network comparing young EVs versus elderly EVs. (A, B) Upregulated disease and function networks predicted by IPA functional enrichment analysis: “Reorganization of cytoskeleton” and “Cell movement.” (C) Downregulated upstream regulator network predicted by IPA functional enrichment analysis: miR‐1‐3p. (D) The IPA network’s legend describes the bubble and line’s colors and the line’s hatchings to better understand the results. In particular, the orange and blue bubbles indicate, respectively, the predicted activation and inhibition of canonical pathways, upstream regulator, and disease and biofunctions; on the other hand, red and green bubbles, respectively, show the increased and decreased protein’s measurement. Purple bubbles represent a specific “protein family name.” The dotted and not dotted network’s lines describe the direct and indirect interaction between molecule–molecule and molecule–function; these lines’ colors indicate a connection with pathways or molecules’ activation (orange lines), inhibition (blue lines), inconsistent findings (yellow lines), and not predicted effects (gray lines), always based on literature data.

The entire protein cargo of EVs samples (156 quantified proteins) was used to perform Gene Ontology and functional enrichment analysis. In this context, Figures 6 and 7 outline the main results obtained by using IPA tool. As previously described, IPA provides insights into the biological pathways involving our protein dataset and, more specifically, into the modulation of these functions. Functional analysis thus offers information on the modulation of functions and the proteins within our dataset involved in this regulation. The networks presented in Figures 6 and 7 highlight the most relevant pathways and the proteins involved in terms of “canonical pathways,” “diseases and biofunctions,” and “upstream regulators.” All proteins shown in these functional networks are listed in Supporting File 3 “protein list” and presented in each graphical network. As shown in Figure 6A,B, EVs isolated from young subjects compared to the elderly ones seem to be involved in a predicted upregulation of “Actin cytoskeleton signaling” (z‐score: 2.324 and –log *p* value: 12.5) and “Integrin signaling” (z‐score: 2.309 and –log *p* value: 7.92); proteins involved in these pathways are presented in the network and listed in the Supporting File 3. The actin cytoskeleton plays an important role in dynamic process such as cell motility; on the other hand, integrins, as cell surface glycoproteins, are involved in cell–cell and cell–extracellular matrix interactions. Since integrins also present interactions with cytoskeletal proteins as actin, this pathway is also involved in cytoskeletal remodeling and again cell motility. Figure 6C explains the IPA network’s legend which describes the bubble and line’s colors and the line’s hatchings. Regarding “diseases and biofunctions,” as downstream biological effect, expected to be modulated, the protein cargo identified in young EVs drives to a predicted activation of “Reorganization of cytoskeleton” (z‐score: 2.748 and *p* value: 1.92E‐08) and “Cell movement” (z‐score: 2.049 and *p* value: 1.05E‐21), compared to the elderly EVs cargo, according to previous results (Figure 7A,B). As shown in Figure 7C, the predicted inhibition of the upstream regulator miR‐1‐3p (z‐score: −1.9 and *p* value: 0.006) in young EVs compared to elderly EVs can explain the observed protein expression changes in the network and is again listed in Supporting File 3. Figure 7D explains the IPA network’s legend which describes the bubble and line’s colors and the line’s hatchings.

### 3.6. Viability and Differentiative Effect of Young EVs on Elderly hMPCs

**FIGURE 8:**
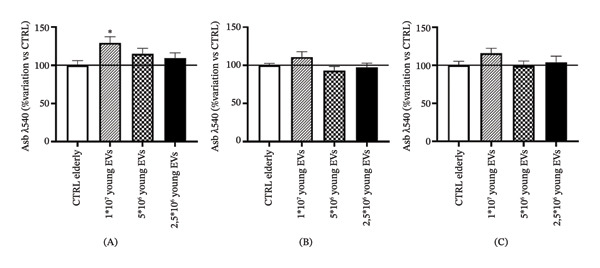
Cell viability of elderly hMPCs stimulated with 1 × 10^7^, 5 × 10^6^, and 2.5 × 10^6^ young EVs. A, B, and C represented the proliferative rate of elderly hMPCs at 24, 48, and 72 h of stimulation with 1 × 10^7^, 5 × 10^6^, and 2.5 × 10^6^ young EVs, respectively. Data are expressed as % variation of stimulated cells vs. CTRL. Three independent experiments were performed. The means ± SEM of *n* = 8 samples were analyzed using unpaired Student’s *t*‐test. ^∗^ *p* < 0.05 vs. CTRL.

**FIGURE 9:**
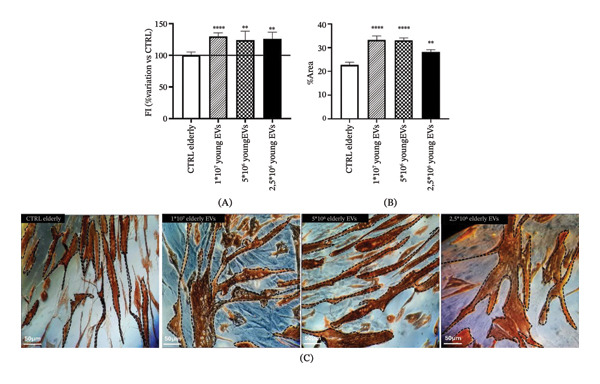
Immunocytochemistry on hMPCs with MF‐20. Fusion index (FI) of elderly myotubes stimulated with 1 × 10^7^, 5 × 10^6^, and 2.5 × 10^6^ of young EVs was calculated. A represented elderly myotubes’ fusion index % at 7 days of differentiation while B showed the % area stained with myosin heavy chain antibody, and C showed representative images with dashed lines to identify myotubes. Data are expressed as % variation of stimulated cells vs. CTRL. The means ± SEM of *n* = 8 samples were analyzed using unpaired Student’s *t*‐test. ^∗∗^ *p* < 0.005; ^∗∗∗∗^ *p* < 0.0001. Scale bar: 50 μm.

We tested the effect of three different doses of young EVs (1 × 10^7^, 5 × 10^6^, and 2.5 × 10^6^) on elderly hMPCs’ viability (Figure 8A–C) and differentiative capacities (Figure 9A–C). The cell viability was assessed using the MTT assay at 24, 48, and 72 h (Figure 8A–C). The MTT assay revealed that 2.5 × 10^6^ and 5 × 10^6^ young EVs did not show any appreciable effect on elderly hMPCs’ viability. Conversely, the highest amount of young EVs (1 × 10^7^) increased the viability of elderly hMPC cultures; this increase was statistically significant at 24 h (^∗^ *p* < 0.05; Figure 8A) while at all other tested time points remained just a trend of increase with respect to control cells.

The differentiation potential of the elderly hMPCs stimulated with 1 × 10^7^, 5 × 10^6^, and 2.5 × 10^6^ young EVs is reported in Figure 9. Here, the FI%, expressed as % variation assessed at 7 days of differentiation, showed a statistically significant increase in the elderly hMPC in presence of all three doses of young EVs with respect to control sample. Images collected after immunocytochemistry assay for myosin heavy chain detection were further analyzed using Immunohistochemistry Image Analysis Toolbox, ImageJ software, showing an increase in the percentage of the stained area in stimulated samples with respect to control, thus confirming the enhanced differentiation process in elderly hMPCs stimulated with young EVs. As shown in Figure 9, elderly myotubes stimulated with young EVs are larger with several myonuclei with respect to control, confirming the increase in the differentiation process.

### 3.7. Viability and Differentiative Effect of Elderly EVs on Young hMPCs

**FIGURE 10:**
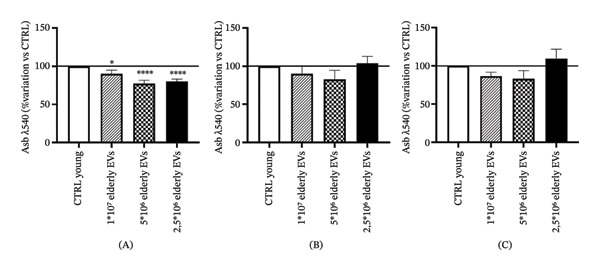
Cell viability of young hMPCs stimulated with 1 × 10^7^, 5 × 10^6^, and 2.5 × 10^6^ elderly EVs. A, B, and C represented the proliferative rate of young hMPCs at 24, 48, and 72 h of stimulation with 1 × 10^7^, 5 × 10^6^, and 2.5 × 10^6^ elderly EVs, respectively. Data are expressed as % variation of stimulated cells vs. CTRL. Three independent experiments were performed. The means ± SEM of *n* = 10 samples were analyzed using unpaired Student’s *t*‐test. ^∗^ *p* < 0.05; ^∗∗∗∗^ *p* < 0.0001 vs. CTRL.

**FIGURE 11:**
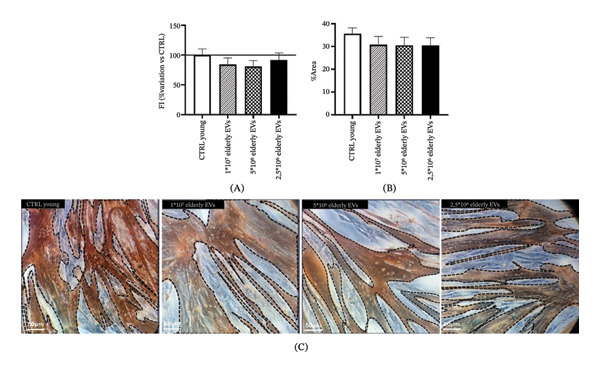
Immunocytochemistry on hMPCs with MF‐20. Fusion index (FI) of young myotubes stimulated with 1 × 10^7^, 5 × 10^6^, and 2.5 × 10^6^ of elderly EVs was calculated. A represented young myotubes’ fusion index percentage at 7 days of differentiation while B showed the % area stained with myosin heavy chain antibody, and C showed representative images with dashed lines to identify myotubes. Data are expressed as % variation of stimulated cells vs. CTRL. Three independent experiments were performed. The means ± SEM of *n* = 10 samples were analyzed. Scale bar: 50 μm.

We tested the effect of the three doses of elderly EVs (1 × 10^7^, 5 × 10^6^, and 2.5 × 10^6^) on young hMPCs’ viability (Figure 10A–C) and differentiative capacities (Figure 11A–C). The cell viability was assessed using the MTT assay at 24, 48, and 72 h (Figure 10A–C). The MTT assay showed that all doses of elderly EVs gave a statistically significant reduction in the viability of young hMPCs cultures at 24 h (^∗^ *p* < 0.05; ^∗∗∗∗^ *p* < 0, 001; Figure 10A), an effect that was not observed at 48 and 72 h.

The differentiation characteristics of the young hMPCs stimulated with 1 × 10^7^, 5 × 10^6^, and 2.5 × 10^6^ elderly EVs are reported in Figure 11. The myotube fusion index of young, expressed as percentage variation, at 7 days of differentiation in presence of all three doses of elderly EVs did not change with respect to control sample. Additionally, images taken for myosin heavy chain detection were analyzed using Immunohistochemistry Image Analysis Toolbox, ImageJ software. Our results demonstrated that the percentage of stained area remained unchanged in stimulated samples compared to the control, suggesting that the differentiation process of young hMPCs is not affected by stimulation with elderly EVs. As shown in Figure 9, young myotubes stimulated with elderly EVs did not change their dimension and contained a similar number of myonuclei with respect to control, confirming the unchanged differentiation capability.

## 4. Discussion

Muscle aging depends on various factors, notably the regenerative capacity of muscle stem cells and their activation into hMPCs. As previously published, it was demonstrated that the in vitro proliferative life span of hMPCs is limited and related to donor age, and their ability to fuse is significantly decreased with age [17]. However, treatment of aged hMPCs with young MPC‐conditioned medium enhanced aged hMPCs’ proliferation and their subsequent differentiation when induced to differentiate [22]. Recently, further evidence showed that skeletal muscle plays a role of secretory organ with both endocrine and paracrine activity, having a major role in this process through the mediation of EVs [12, 33]. Moreover, in our previous study, we have isolated and characterized the EVs demonstrating that young hMPCs release EVs stuffed with guanosine‐based molecules [26]. In the present work, we demonstrated that the impaired differentiation process of elderly hMPCs is rescued by exposure to young EVs.

This suggests that hMPCs, much like skeletal muscle, secrete bioactive factors and signaling molecules via EVs.

This prompted us to analyze the cargo of EVs released by hMPCs as proteins and miRNAs and to evaluate the specific contribution of EVs released by young hMPCs in culture media as a stimulus capable of modulating the vitality and differentiation potential of elderly hMPCs and vice versa.

EVs collected from young and elderly subjects exhibited comparable average sizes and particle concentrations, as confirmed by physicochemical analysis. The average sizes of different samples had a consistent intra‐ and inter‐sample variability for all EV samples at the similar average sizes of ∼ 270 nm, with a heterogeneous size distribution ranging between 100 and 450 nm, which may be due to the intrinsic variability of patients‐derived cells [34]. Indeed, the average sizes and particle concentration of EVs, collected in our samples, also depended on the isolation method that is used to collect samples. Ultracentrifugation is a reliable method for EV isolation [26, 35], which is a solid and valid method approved to isolate and collect EVs, which limited the narrow size distribution of EVs collected from multiple subjects [36] that have high variability and composition in the same culture of hMPCs where EVs came from.

Moreover, the size spectrum observed in elderly subjects, compared to the distribution in young subjects, could suggest a loss of control in vesicular biogenesis and an accumulation of debris resulting from inflammaging, which reduces the distinction between EV subpopulations [37].

Moreover, in the literature, it is known that variability could also depend on contaminants in EV samples such as protein aggregates, RNA–protein complexes, and lipoproteins, which resemble EVs in shape, size, and/or density. These contaminants, originating from body tissues and cellular membranes, are not easily removed by using ultracentrifuge and contributed to the intra‐ and inter‐sample variability of EVs that are distributed in the range from 80 to 500 nm, result similar to that measured for our samples collected from young and elderly subjects [38]. Variability due to contaminants may explain the particle concentrations observed in all analyzed samples. In fact, the variability of average sizes and particle concentration of EVs can influence their signaling responsiveness in the recipient cells and their intracellular communication network [39]. Nevertheless, EVs are key mediators of molecular transport and intercellular communication. Therefore, considering this variability, we verified by WB for the tetraspanin CD81, characteristic exosome marker [35, 40], in order to identify our isolated EVs. Further characterization of exosomes in our samples confirmed the presence of ALIX, exosome marker [35]. Indeed, as reported in our results, young and old EVs exhibited ALIX expression in immunofluorescence. Moreover, as reported in the literature, ALIX is essential for characterization of EVs as well as plays a role in miRNA packaging during EV biogenesis [41]. Therefore, we have also focused our attention on the study of miRNAs in EVs [42]. These results highlight the intra‐sample variability discussed above, confirming the presence of EVs as important players in cell‐to‐cell communication in normal physiological conditions [43].

We specifically investigated the molecular cargo of EVs, focusing on the expression of muscle‐specific miRNAs called myomiRs. Notably miR‐1, miR‐206, miR‐133a, and miR‐133b, are essential for orchestrating the proliferation and differentiation of skeletal muscle [19, 26]. In detail, miR‐1 and miR‐206 are key myomiRs for Mef2c‐dependent terminal myogenic differentiation. miR‐1 and miR‐206 act as positive controls for the progression of differentiation via Notch3 and utrophin inhibition [44, 45]. Among the various myomiRs, miR‐133 a and b are involved in the modulation of muscle growth and differentiation [8]. In our data, miR‐133 a and b showed a trend of upregulation while miR‐1 and miR‐206 showed a trend of downregulation in young EVs versus elderly ones. Assuming that the EVs possess a functional cargo in part representative of the cells of origin [46, 47], we compared present data on myomiR expression on EVs with our previous study on myomiR expression on hMPCs [19]. The comparison highlighted that miR‐1 has a trend of downregulation in young samples with respect to elderly samples both in EVs and hMPCs; this result is also in line with the prediction made by IPA software in proteomic analysis. It was demonstrated that miR‐1 is correlated with an increased apoptosis via heat shock protein 60 and heat shock protein 70 in elderly hMPCs in proliferative condition [19]. Therefore, the downregulation of miR‐1 together with the upregulation of heat shock protein found in proteomic analysis in our samples of young EVs further confirms that young cells are less affected by apoptosis. This data crossover represents a strong point as it is a mechanistic confirmation between proteomic and miRNA analyses. These data are also in line with previous findings demonstrating that a lower proportion of young hMPCs undergoes spontaneous apoptosis compared with elderly hMPCs [18]. In the literature, it is known that miR‐206 is more expressed during myogenesis [48, 49], a process more efficient in the muscle of young subjects compared to that of elderly subjects. But surprisingly, data collected both on hMPCs [19] and EVs seem to be in contrast with the literature. Indeed, in a previous work comparing young and elderly hMPCs during both proliferation and differentiation, no difference in miR‐206 expression was found [19].

Here, we found no significant differences in the miRNA content of proliferating hMPC‐derived EVs, although miR‐206 showed a trend of downregulation in the expression of miR‐206 in young EVs compared to elderly ones. The reduced expression of miR‐206 in young EVs may stem from preferential intracellular retention, suggesting that cells prioritize endogenous utilization over extracellular release [47, 50, 51]. Anyway, beyond the comparison between young and elderly EVs, the young EVs in this work were used as a stimulus on the elderly hMPCs carrying miR‐206 in their cargo together with proteins and other not investigated factors, still able to induce effects on proliferative and differentiative capacity on elderly hMPCs, as discussed below. Furthermore, it must be noted that the amount of miRNA is related and can vary depending on the method with which the EVs are isolated, as demonstrated by Wang et al. [52]. In the literature, it is known that miR‐133a and miR‐133b are involved in myoblasts proliferation and are downregulated in skeletal muscle during aging [19, 53]. Accordingly, we observed in our samples a trend of downregulation of miR‐133a and miR‐133b in elderly EVs with respect to young ones. Moreover, miR‐133a and miR‐133b are involved in sarcomeric actin organization in accordance with our proteomic data [48, 54]. Indeed, the analysis of the proteomic cargo of EVs using IPA software highlights significant differences in the biological pathways involved between EVs from young and elderly subjects [55, 56]. Specifically, EVs from young subjects appear to be particularly involved in actin cytoskeleton signaling and integrin signaling pathways known for their role in cellular motility and cell–cell and cell–extracellular matrix interactions [57, 58]. Indeed, adhesion molecules and the numerous signaling cascades strictly rely on myogenesis [53]. These results suggest that EVs from young subjects may have a more active role in modulating cellular dynamics and interaction with the surrounding environment compared to elderly EVs. Given the miRNA and proteomic cargo so far discussed, we firstly investigated the effects of young EVs on vitality and regenerative potential of elderly hMPCs. To the purpose, three different amounts of young EVs were used (1 × 10^7^, 5 × 10^6^, and 2.5 × 10^6^) assuming that they are similar to the physiological range released by our cells (as shown in Table 1) [59]. While the effect of young EVs on elderly hMPCs’ viability gave a statistically significant increase only at the highest amount at 24 h (and a trend of increase at 48 and 72 h). Surprisingly, the capacity of cells to fuse and mimic the *in vitro* regenerative process was significantly enhanced in stimulated elderly cells relative to the control group, irrespective of the dose of young EVs administered. Indeed, this analysis showed that the cargo of young EVs resulted in restoration of the differentiation potential. Conversely, stimulation with elderly EVs significantly decreased young hMPCs’ viability at early time point (24 h), but then (at 48 and 72 h) they recovered the replicative capacity. Even the differentiative property of young hMPCs was not affected by the stimulation with elderly EVs, confirming that the intrinsic resilience of young hMPCs allows them to rapidly overcome the initial negative effect of elderly EVs. This observation is in line with several previous publications demonstrating the ability of young hMPCs to rescue by an oxidant insult, to properly differentiate in vitro and maintain an efficient EC coupling mechanism with respect to elderly hMPCs [16, 17, 19].

Overall, our data show that miRNAs and protein cargo of young EVs enhance the differentiation process preceded by a proliferative boost, as confirmed by the increase in viability rate on elderly hMPCs. The input given by young EVs on the myogenic process is in accordance with proteomic analysis, which has revealed an increase in proteins involved in the cellular movement pathway in young EVs compared to the elderly EVs. On the other hand, elderly EVs did not alter young hMPCs’ behavior in response to external stimuli due to their intrinsic properties.

## 5. Conclusion

Muscle stem cell–derived EVs act as key intercellular vehicles, orchestrating the delivery of bioactive signaling molecules. Our results demonstrate that elderly hMPCs‐derived EVs impair the regeneration of young hMPCs. Indeed, we demonstrated that elderly hMPCs‐derived EVs exert a negative impact by impairing the regeneration process in young hMPCs.

Strikingly, the EVs released by young hMPCs carry regenerative signals that reverse the functional decline of aged muscle stem cells. Here, we showed the potential of EV cargo from young hMPCs to enhance the proliferative and differentiation capacity of hMPCs derived from elderly subjects, giving cells a rejuvenating effect.

This study corroborates recent findings that identify skeletal muscle as a secretory organ whose endocrine and paracrine activities are also modulated by EVs.

In conclusion, our findings provide new insights for future research into the molecular mechanisms underlying EV–cell interactions.

## 6. Limitations of the Study

One of the limitations of the study is the use of only male subjects. It would be interesting to also evaluate the impact of EVs derived from female subjects. Indeed, also analyzing the EVs derived from female subjects can enable more in‐depth study to describe the cargo of the EVs and the effects on hMPCs, also due to different hormonal modulations between men and women. Indeed, it is known that different hormonal compositions produce different results in response to muscle regeneration. Another limitation of the present study was the difficulty in recruiting a higher number of volunteers. Furthermore, in this study, we worked with primary cells that exhibit replicative senescence, and so this limits the capacity for large‐scale in vitro expansion as well as the interpersonal variability of individual subjects which can lead to data variability.

## Author Contributions

Lorenzo Marramiero: data curation, formal analysis, investigation, methodology, validation, and writing–original draft. Ester Sara Di Filippo: data curation, formal analysis, investigation, methodology, validation, writing–original draft, and writing–review and editing. Federica Di Marco: data curation, formal analysis, investigation, methodology, and validation. Piero Del Boccio: methodology, conceptualization, and writing–original draft. Cristian Celia: methodology, conceptualization, resources, visualization, and writing–original draft. Luisa Di Marzio: methodology, conceptualization, resources, visualization, and writing–original draft. Nicola d’Avanzo: data curation, formal analysis, investigation, methodology, and validation. Tiziana Pietrangelo: conceptualization, visualization, and writing–review and editing. Stefania Fulle: conceptualization, funding acquisition, resources, visualization, and writing–review and editing. Rosa Mancinelli: conceptualization, project administration, supervision, visualization, and writing–review and editing.

Lorenzo Marramiero and Ester Sara Di Filippo contributed equally.

## Funding

This research was funded by Ministero dell‘Università e della Ricerca’ (MUR) to Stefania Fulle, Christian Celia and Luisa Di Marzio: the European Union – NextGenerationEU, under the National Recovery and Resilience Plan (NRRP), Mission 4 Component 2 ‐ M4C2, Investment 1.5 – Call for tender No. 3277 of 30.12.2021, Italian Ministry of University, Award Number: ECS00000041, Project Title: “Innovation, digitalization and sustainability for the diffused economy in Central Italy”, Concession Degree No. 1057 of 23.06.2022 adopted by the Italian Ministry of University. CUP: D73C22000840006. This study was also supported by local grants of the University ‘G. d’Annunzio’ of Chieti‐Pescara [FAR 2018 (D56C18000780005), FAR 2019 (D54I19002790005)] to Christian Celia and Luisa Di Marzio.

Open‐access publishing was facilitated by Universita degli Studi Gabriele d’Annunzio Chieti Pescara, as part of the Wiley–CRUI‐CARE agreement.

## Disclosure

All authors agreed to publish the study.

## Ethics Statement

This study was conducted according to the Helsinki Declaration (as amended in 2000) and approved by Ethics Committee of the Provinces of Chieti and Pescara (prot.n. 16/2019).

## Consent

All the subjects provided their written informed consent.

## Conflicts of Interest

The authors declare no conflicts of interest.

## Supporting Information

Additional supporting information can be found online in the Supporting Information section.

## Supporting information


**Supporting Information 1** Supporting File 1. Excel file contains the list of the quantified proteins “canonical pathways,” “upstream regulators,” and “downstreams” generated by the IPA system.

**Supporting Information 2** Supporting File 2. Excel file “Proteomics report” in purified EVs from proliferating cell culture media of young and elderly subjects contains the list of the quantified proteins common in at least two of the analyzed replicates (*n* = 3).

**Supporting Information 3** Supporting File 3. Excel file contains protein list of “Actin cytoskeleton signaling,” “Integrin signaling,” “Reorganization of cytoskeleton,” “Cell movement,” and “miR‐1‐3p” pathways in the EVs isolated from young subjects compared to the elderly ones.

## Data Availability

Data will be made available upon request. Proteomic data are available via ProteomeXchange with identifier PXD063794.
